# Revisiting HPV vaccination post-COVID: geopolitical, sociocultural, and ethical disparities in global health

**DOI:** 10.1186/s12939-025-02669-y

**Published:** 2025-11-10

**Authors:** Sondos Al Sad, Labiqah Iftikhar, Masa Chamout

**Affiliations:** 1 Family and Community Medicine Department, Women’s Health Primary Care Center, University of California San Francisco (UCSF), 2356 Sutter St., San Francisco, CA 94115 USA; 2 School of Public Health Sciences, University of Waterloo, 200 University Ave W, Waterloo, ON N2L 3G1 Canada; 3 Faculty of Science, University of Waterloo, 200 University Ave W, Waterloo, ON N2L 3G1 Canada

**Keywords:** Indigenous health, Religiosity, Cervical cancer, Health equity, Healthcare discrimination

## Abstract

**Background:**

HPV vaccines have been revolutionary in preventing HPV-related cervical cancer and reshaping the cervical cancer screening guidelines in the past decades. Yet, challenges persist in achieving universal accessibility and utilization. Since the COVID-19 pandemic, shifts have emerged in HPV vaccine research, implementation strategies, and the determinants shaping uptake and delivery, particularly from a global equity perspective.

**Methods:**

This is a scoping review examining English-language, peer-reviewed articles published following the onset of the COVID-19 pandemic until the end of 2024. It focuses on the human papillomavirus (HPV) vaccine and factors influencing its uptake. Articles were retrieved from PubMed and Embase databases and screened for relevance using predefined search terms.

**Results:**

Out of 2755 articles, 349 were included. We identified that most peer-reviewed articles focus on interventions and implementation strategies more than acknowledging geopolitical affairs, gender specificity, religious and ethical dimensions, medical mistrust, or healthcare discrimination. Most of the articles were cross-sectional in nature and most were funded by the National Cancer Institute. Interestingly, we found no peer-reviewed articles on the intersectionality of Judaism and HPV vaccine uptake, with a limited number on Islamic, Christian, or other religious intersectionality. Articles addressing how low- and middle-income countries could be equipped to develop and manage their own vaccine programs and manufacturing were largely absent; instead, cost-effectiveness research focused primarily on the vaccine’s ability to reduce disease burden.

**Conclusion:**

Post-pandemic research on HPV vaccination indicates that levels of hesitancy and uptake have remained relatively stable. However, the literature highlights persistent inconsistencies in how the vaccine is prioritized across communities, healthcare professionals, and health systems. Messaging regarding its importance for cancer prevention remains fragmented, while cost barriers and the absence of the vaccine from many national immunization schedules continue to limit access. Notably, ethical, religious, and cultural considerations receive limited attention in current research, despite the pandemic underscoring the global significance of these factors in shaping health behaviors. These findings suggest a need to re-examine how HPV vaccination is framed and advanced as a public health priority within diverse sociocultural and systemic contexts.

## Introduction

Human Papillomavirus (HPV) is a circular DNA virus transmitted primarily through intimate sexual contact, with rare reports of non-sexual transmission [1]. Its clinical impact ranges from asymptomatic to causing cancer in many human organs [2]. The Centers for Disease Control (CDC) confirms that approximately 80% of women and 90% of sexually active men will acquire HPV during their lifetime [3]. While most HPV infections are asymptomatic and clear spontaneously within 6–24 months (9.4 months for cervical HPV in women and 7.5 months for genital HPV in men) [4], persistent infection with high-risk types can progress to warts within 6–10 months and, over 5–25 years, to cancers, including cervical, anogenital, and oropharyngeal malignancies [5]. Carcinogenic HPV types (16,18, 31, 33, 35, 39, 45, 51, 52, 56, 58, and 59) [6] account for roughly 9% of cancers in women and 1% in men [7]. Host susceptibility (e.g. immunocompetence, hormonal profile, age) [8, 9], alongside behavioral and contextual factors such as smoking, stress, health literacy, healthcare access, and vaccination status [10, 11] influences persistence and cancer development. Although HPV pathogenesis and its progression to cancer are well established, our review highlights that knowledge gaps persist across training and practice levels—from medical students to specialists. This gap is particularly evident regarding HPV transmission, clearance, and vaccine-preventable cancer risks, suggesting a critical need for more consistent education to align clinical understanding with evidence-based public health priorities.

HPV vaccines are most effective in preventing HPV infection in both females (43–100%) and males (25–100%) younger than 26 years of age, with minimal side effects [12–14]. The limited available literature indicates that these vaccines are similarly effective in women up to 45 years of age [15, 16]. Since the introduction of the HPV vaccine programs in the US in 2006, a significant decrease in the burden of most HPV-related cancers and even genital warts has been reported (see Table 1) [27].

**Table 1:** HPV-related pathologies and impact of HPV vaccine on its burden

Pathology	Burden ASR [17] ^(1)^ (incidence/mortality)	HPV association [18] ^(3)^	Impact of HPV vaccine (Reported impact%)
Cervical cancer	14.1/7.1	91%	53–87% [19] ^(6)^
Vaginal cancer	0.36/0.15	75%	84% [20] ^(7)^
Vulvar cancer	0.83/0.30	69–86% [21] ^(4)^	77–95% [22] ^(8)^
Penile cancer	0.79/0.28	63%	NA
Anal cancer	0.54/0.21	> 90%	77.5% [23] ^(9)^
Oropharyngeal cancer	1.1/0.53	70%	80%
Genital warts (HPV 6 &11)	120.5 [24] ^(2)^/NA	> 90% [25] ^(5)^	73% [26] ^(10)^-97%

HPV: Human Papilloma Virus, ASR: age-standardized rate per 100,000 persons-year, NA: not applicable, (1: worldwide, 2: worldwide, 3: USA, 4: Denmark, 5: Turkey, 6: Denmark, Sweden, UK, 7: Denmark, 8: worldwide, 9: Australia, Brazil, Canada, Croatia, Germany, Spain, United States, 10: Spain)

The focus of HPV vaccine research centered on women due to the well-established link between HPV and cervical cancer, with the cervix serving as a proxy endpoint for assessing vaccine efficacy. Unlike cervical cancer, there was no comparably prevalent cancer in males with well-defined clinical endpoints [28], and while genital warts affect both genders, initial vaccine trials primarily involved females. The primary driver behind vaccine development was the prevention of cancer [12]. Thus, the vaccine was initially approved for adolescent females based on available data and the societal context at the time of its discovery. This led to a gendered perception of the HPV vaccine and resulted in a significant gap in its administration to eligible males [29].

Nonetheless, HPV is transmitted through sexual contact, including skin-to-skin, genital-to-skin, and oral-to-genital contact. Research indicates that nearly 85% of women and 91% of men with at least one opposite-sex sexual partner will contract HPV at some point in their lives [30]. Furthermore, males are more likely to acquire HPV and act as recipients of the virus in heterosexual transmission scenarios. These findings challenge the notion that HPV is solely a female-specific infection [31].

The oncogenic impact of HPV infections is generally more pronounced in females when examining all cancers without stratification. However, a global overview reveals regional differences in male HPV-related cancer incidence and specific cancer types. For example, HPV-related cancers are increasingly prevalent among males in the Global North compared to females, underscoring the need for gender-neutral vaccination campaigns in these regions. Additionally, the incidence rate of oropharyngeal HPV-related cancers is higher in males than in females.

Even though the HPV vaccine has demonstrated its preventive effectiveness, cost efficiency, and low risks, it is not uniformly accessible or sufficiently utilized worldwide [32, 33]. In a recent review, system-level factors and community-level factors were listed as potential influencers of HPV vaccine program introduction and implementation [34]. System-level encompassed financial considerations, vaccine prioritization, global supply, capacity, and delivery capabilities, as well as considerations related to vaccine accessibility, equity, and ethics. Whereas, community-level factors included vaccine acceptability and hesitancy, as well as communication strategies, advocacy efforts, and social mobilization within the community [34]. Many studies pointed out the lack of provider’s recommendation as a main and common barrier to getting the HPV vaccine regardless of patient’s demographics (i.e. gender, language, ethnicity, etc.) [35, 36].

The COVID-19 pandemic introduced new challenges to an already inequitable landscape of HPV vaccine uptake, intensifying long-standing issues such as vaccine hesitancy, medical mistrust, and disparities in healthcare access. Prior to the pandemic, the World Health Organization (WHO) had identified vaccine hesitancy as one of the top 10 global health threats, with specific concerns surrounding the comparatively lower uptake of the HPV vaccine [37]. The pandemic disrupted routine healthcare services, further marginalized vulnerable communities [38], and heightened vaccine hesitancy, as healthcare systems prioritized COVID-19 responses over essential preventive care, including vaccinations [39]. This reprioritization was accompanied by rising public skepticism about vaccines, with distrust of the COVID-19 vaccine extending to other immunizations, including the HPV vaccine. The compounded effects of missed healthcare appointments, shifting public health priorities, and increased hesitancy merit the need for a thorough evaluation of the pandemic’s impact on HPV vaccine uptake in a post-pandemic context.

In contrast to prior reviews that emphasized inequities in HPV vaccination through geopolitical, sociocultural, and ethical lenses, our scoping review sought to determine whether the COVID-19 pandemic shifted how HPV vaccination and the related pathologies are studied. We identified a uniform pattern in the literature: the dominant focus remained on low vaccine uptake, while little attention was directed toward HPV-related pathologies themselves or how pandemic conditions may have altered their prioritization. This gap is compounded by limited research on religious or ethical dimensions of vaccine acceptance, underrepresentation of data from low-income countries, and minimal exploration of healthcare discrimination and mistrust within HPV programming. By interrogating these gaps, our review aims to understand whether the pandemic has meaningfully reshaped perspectives on an elective vaccine such as HPV, or whether existing approaches continue to overlook the cultural and contextual uniqueness that shapes health outcomes worldwide.

## Methods

### Literature search

A scoping literature review was conducted to investigate the multifaceted dynamics of preventive strategies, and the religious, ethical, and sociocultural determinants influencing HPV vaccine uptake and healthcare delivery in the geopolitical context following the COVID-19 pandemic. We searched PubMed and Embase for peer-reviewed articles written in or translated into English, with data collected after March 2020 or explicitly referencing the COVID-19 pandemic, up to the end of 2024. See Table 2 for detailed inclusion and exclusion criteria.

**Table 2:** Inclusion and exclusion criteria

Inclusion Criteria	Exclusion Criteria
Peer-reviewed articles	Non-peer-reviewed articles, reviews, editorials, book chapters, and opinion pieces
Published in English	Articles in languages other than English
Publications from 2021 onwards (or data collected after March 2020)	Excluded preprints
Studies focusing on HPV vaccine uptake, efficacy, and barriers	Studies not relevant to the HPV vaccine
Articles discussing sociocultural, geopolitical, and sociodemographic factors influencing vaccine implementation and dissemination	

We searched PubMed and Embase for studies with data collected between March 2020 (the onset of the COVID-19 pandemic) and December 31, 2024. The search strategy combined controlled vocabulary (MeSH for PubMed and Emtree for Embase) with free-text terms, focusing on HPV vaccination in relation to religious affiliation, cultural factors, political context, and health equity–related determinants. Controlled vocabulary terms were explored when appropriate to capture all narrower terms. Full search strings for both databases are provided in the Appendix to ensure reproducibility.

Search results were imported into Covidence systematic review software [40], which automatically removed duplicates and facilitated the screening process. Three authors independently reviewed titles, abstracts, and full texts against predefined inclusion criteria. Any discrepancies were resolved through discussion until consensus was reached. Articles meeting the inclusion criteria were retained for the final synthesis. The authors then extracted relevant data and analyzed the content to identify recurring themes, which were inferred in alignment with the study’s aims.

## Results

**Fig. 1:**
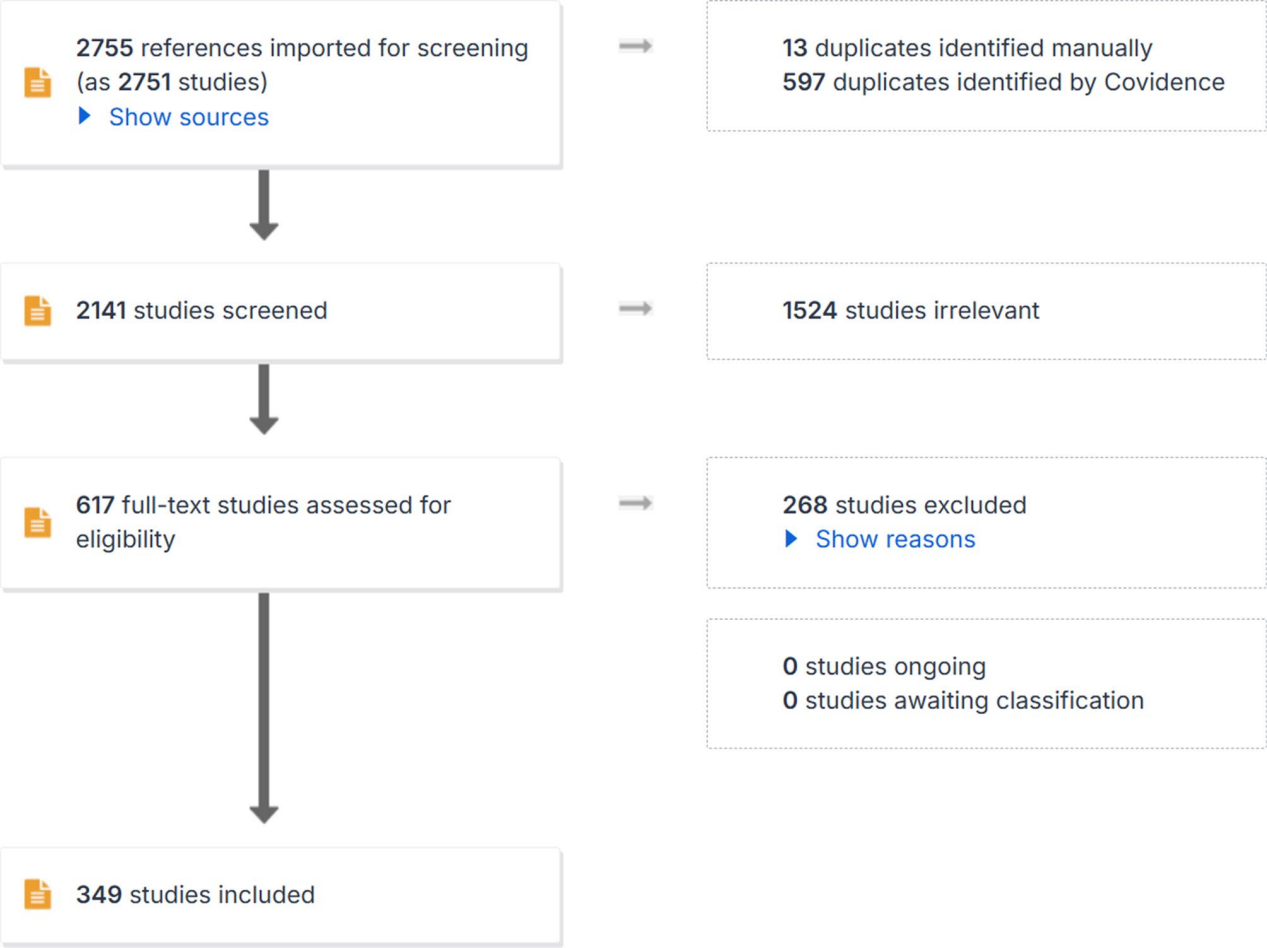
PRISMA diagram outlining screening on Covidence

The initial search yielded 2755 peer-reviewed articles, published post COVID-19 pandemic about HPV vaccine intake and search words. After removing duplicates and applying the exclusion criteria, 349 articles remained for further analysis (See Fig. 1). From the selected articles, relevant data were extracted including funding, population characteristics, geographic location, key findings, and identified barriers and facilitators to HPV vaccine uptake.

The articles reviewed encompassed qualitative, quantitative, and mixed-methods studies, with the majority being cross-sectional surveys (62%). In total, 73 countries were represented (top 5: USA, China, Turkey, Saudia Arabia, and Ethiopia), and 218 studies reported funding, primarily from the National Cancer Institute (NCI), National Institutes of Health (NIH), and Merck Sharp & Dohme, a U.S.-based multinational pharmaceutical company. Only 181 articles referenced the COVID-19 pandemic or COVID-19 vaccines in their work. Religion was addressed in only a handful of studies, with references limited to Muslims (2) and Christians (4); none explicitly discussed Judaism, apart from indirect mention in studies conducted in Israel (3).

The extracted data were synthesized using a narrative review approach. Key themes were identified and categorized under major headings: Political determinants of health, Sociodemographic characteristics, and Provider’s characteristics. Most articles addressed more than one theme; Table 3 provides a summary of the number of articles focused on each key theme. These themes have been discussed in the context of their impact on HPV vaccine uptake and healthcare delivery.

**Table 3:** Classification of articles by theme and sub-themes

Themes	No. of Articles
Political determinants of health	345
Sociodemographic characteristics	261
Provider’s characteristics	116

Note that there were overlapping themes present throughout the articles. The theme “Sociodemographic characteristics” includes the patient and healthcare provider population

## Key themes

### Political determinants of health

#### Geopolitical affairs

**Fig. 2:**
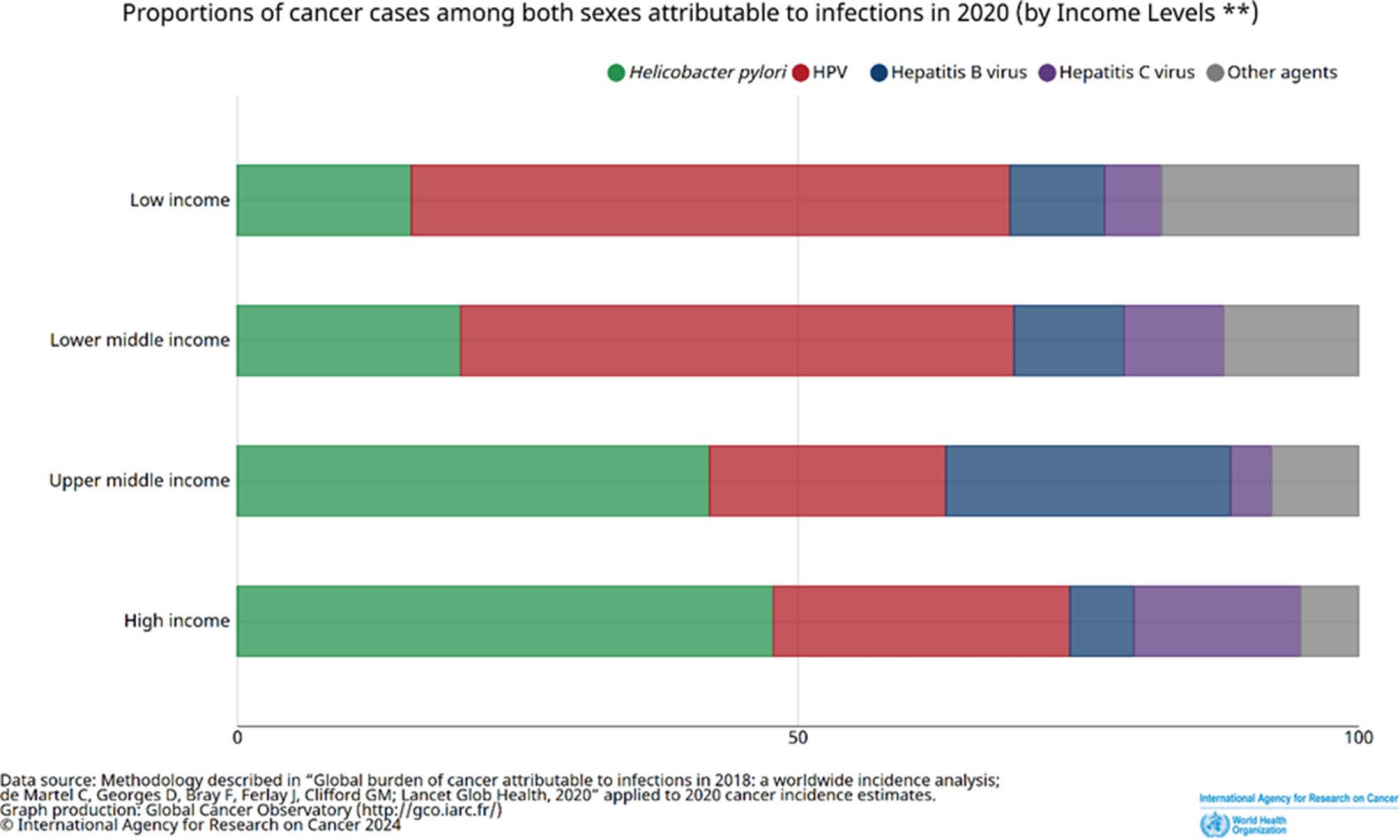
Proportions of HPV-related cancers stratified by country’s income level. World Health Organization 2020. Data are from the GLOBOCAN database, collated by the International Agency for Research on Cancer and hosted by the Global Cancer Observatory

The COVID-19 pandemic significantly reshaped geopolitical dynamics, influencing vaccine availability, manufacturing, and patterns of hesitancy [34]. This influence becomes evident when examining global disparities in vaccine access and utilization [41]. In the case of HPV vaccination, despite low- and middle-income countries bearing a significant burden of cervical cancer cases, there is a glaring lack of targeted HPV vaccination programs in these regions (see Fig. 2) [42]. In 2020, only 41% of low- and middle-income countries had introduced HPV vaccination compared with 88% of high-income countries [41]. Vaccination coverage significantly declined in low- and middle-income countries, with a 17% point drop in low-income countries and an 11% point drop in middle-income countries in 2020 [43], causing many girls to age out of the 9–14 year-old target vaccination group [44]. Hence, there is an effort to introduce and investigate a one-dose efficacious HPV vaccine program in several low- and middle-income countries to mitigate the scarcity of resources and cost challenges [43]. While disparities between high- and low- to middle-income countries are well-documented, empirical data on fully implemented national HPV vaccine programs remain limited. Modeling studies suggest that cost-effective vaccine strategies could yield substantial public health benefits in countries such as Ghana [45], indicating that implementation at this scale is feasible and economically justified. Additionally, smaller-scale initiatives, such as a school-based pilot in Nigeria [46], demonstrate the potential for local adaptations to increase access and uptake in resource-limited settings. These examples highlight how geopolitical and economic contexts shape the feasibility and design of HPV vaccination efforts, even when comprehensive program-level data are not yet available.

The COVID-19 pandemic has further underscored the geopolitical complexities surrounding COVID-19 vaccine manufacturing and distribution [47]. Wealthier nations with the financial means often secure agreements to monopolize vaccine production, leaving poorer countries at a disadvantage in accessing life-saving immunization [48]. This disparity reinforces existing global power dynamics and raises ethical concerns about equitable vaccine distribution in times of crisis [49]. Country income did not consistently determine HPV vaccine cost coverage.

Instead, factors such as vaccine price, number of doses required, availability, whether vaccination was mandated or elective, age of eligible candidates, and cultural context more strongly influenced uptake and implementation [34]. Income appeared to function more as an indirect proxy for how countries adopt HPV vaccine narratives and how they might be empowered to manufacture vaccines suited to their populations’ needs [50].

Overall, our findings suggest that vaccine uptake is shaped by complexities that extend beyond national economic status [51, 52]. HPV vaccine uptake in low- and middle-income countries (LMICs) remains shaped by structural inequities amplified in the post-COVID-19 context [53]. The pandemic deepened global disparities in vaccine access, as high-income countries secured supply while LMICs faced shortages and delayed rollouts [54, 55]. These inequities extend to HPV vaccination, which is absent from many national immunization schedules due to cost barriers, competing health priorities, and constrained fiscal capacity [56, 57]. Political unrest and economic hardship have further strained health systems, undermining trust and limiting governments’ ability to prioritize preventive measures such as HPV vaccination [56, 58].

The neglect of sociocultural dimensions—including ethics, religion, and gender norms—within global health policy adds complexity to uptake, despite evidence that culturally informed approaches are essential for acceptance [59]. The introduction of vaccines like HPV is therefore not solely driven by medical necessity, but also by political agendas and global power dynamics [60]. Although global supply is currently sufficient, WHO cautions that access constraints may persist over the next three years due to limited supply buffers post-pandemic, underscoring the need for gender-neutral and catch-up campaigns and flexibility in vaccine choice to reduce shortages.

There are 5 types of HPV vaccines [61] that are deemed equally effective by WHO [62, 63] (See Tables 4 and 5).

**Table 4:** Current HPV vaccines have received marketing authorization and/or WHO prequalification

Vaccine Type	Target Cohort	Manufacturer	Ingredients [64]	Frequency of doses [65]	Type of HPV targeted
Bivalent (HPV2)	Girls and women, boys and men (9–45 years)	GSK (Cervarix®)	AS04 adjuvant (expression system: Insect cell)	Girls and boys aged 9–14 years on as 2dose schedule (5–13 months apart). From age 15, three doses should be given (at 0, 1–2.5 months and 5–12 months)	HPV Type 16 and 18 [66]
Bivalent (HPV2)	Girls and women (9–45 years)	Innovax (Cecolin®)	Aluminium-containing adjuvant (produced in a bacteria extracted from mammalian cell outside the vaccine production system)	Girls aged 9–14 years as 2dose schedule (6 months apart). From age 15, three dose schedule is indicated (at 0, 1–2 months and 5–8 months)	HPV Type 16 and 18 l [63]
Bivalent (HPV2)	Girls and women (9–30 years)	Walrinvax™ Biotechnology (Shanghai Zerun Biotech)	Aluminium-containing adjuvant (Expression system: Yeast)	Girls aged 9–14 years as 2dose schedule (6 months apart, with a minimum interval of 5 months). From age 15, threedose schedule is indicated (at 0, 2–3 and 6–7 months)	HPV Type 16 and 18 [67]
Quadrivalent (HPV4)	Girls and women, boys and men (9–45 years)	Merck (Gardasil®)	Aluminium-containing adjuvant (expression system: yeast)	Girls and boys aged 9–13 years as 2dose schedule (6 months apart). From age 14, threedose schedule should be given (at 0, 1–2 and 4–6 months)	HPV Types 6, 11, 16, 18 [68]
Non-valent (HPV9)	Girls and women, boys and men (9–45 years)	Merck (Gardasil 9®)	Aluminium-containing adjuvant (expression system: yeast)	Girls and boys aged 9–14 years, as 2dose schedule (6 months apart). From age 15, threedose schedule should be given (at 0, 2 and 6 months)	HPV Types 6, 11, 16, 18, 31, 33, 45, 52, and 58 [68]

**Table 5:** HPV self-procured price per dose depending on countries’ income level (2021)

Income Level	Vaccine Type	Price Per Dose (USD)
Upper-Middle-income countries	HPV2	$13–$11
	HPV4	$64 - $39
	HPV9	$14
High-income countries	HPV2	$27 - $25
	HPV4	$149 - $126
	HPV9	$165 - $101
Countries within US Centers for Disease Control and Prevention	HPV9	$165
Countries with PAHO (Pan American Health Organization) Revolving Fund (RF) divisions	HPV9	$9.98
Countries with UNICEF (United Nations Children’s Fund) Supply Division (SD)	HPV9	$4.50

Tobacco regulation is another significant geopolitical matter, yet under-researched policy factor in the global burden of HPV-related cancers, especially in low- and middle-income countries (LMICs) where public health initiatives are less robust. Tobacco use increases the risk of persistent HPV infection, leading to higher incidences of vaginal, vulvar, anal, and oropharyngeal cancers [69, 70]. For instance, women treated for precancerous lesions who smoke are substantially more likely to experience cancer progression than nonsmokers [71, 72]. Additionally, while high-income countries with strong tobacco control policies have seen decreases in certain cancer types, such as lung cancer [73], the impact of these regulations varies globally, influenced by disparities in tobacco consumption, healthcare access, and HPV screening programs. Therefore, the intersection of tobacco regulation and HPV vaccination efforts could significantly influence cancer outcomes, but remains an area requiring more comprehensive research, particularly in regions where tobacco control lags behind [74].

#### Healthcare discrimination, medical mistrust, and media

Healthcare discrimination—rooted in structural racism, lack of diverse representation, and historical injustices—contributes significantly to medical mistrust and vaccine hesitancy. Medical mistrust, often shaped by past and ongoing discrimination, reflects reluctance to trust healthcare systems perceived as aligned with dominant culture, and can manifest as a defensive response even when not directly experienced [75–79]. Studies have consistently shown that foreign-born individuals, Black and Latina populations, people of color, those with lower incomes, and non-binary individuals are disproportionately affected by healthcare discrimination and are more likely to express mistrust in healthcare systems [80–84]. These perceptions must be analyzed through a structural lens, highlighting the need for systemic changes alongside community-driven interventions. In relation to HPV vaccine uptake, research has shown that although barriers to access do affect uptake in minority groups such as Black and Latino adolescents and parents, medical mistrust is a greater factor influencing vaccine acceptance [85]. This goes to show the importance of identifying trusted sources to target communities in need of intervention.

A key barrier to HPV vaccination in marginalized populations is mistrust in government and pharmaceutical companies, stemming from historic injustices. Such as Tuskegee Syphilis Study [86, 87]. Historical traumas can carry on for generations and influence the delicate fabric of trust between minority groups and the healthcare system, specifically in relation to the HPV vaccine.

Media has emerged as an important geopolitical determinant of HPV vaccine dynamics, particularly in the post-pandemic context [88]. Web-based surveys [89] and virtual interviews have expanded research methodologies, while public health campaigns have increasingly relied on digital platforms [90], including online advertisements, campus-based virtual modules [91], and social media outreach [92]. Studies have also examined the influence of social media content on vaccine uptake and hesitancy [93], highlighting its amplified role during and after the pandemic. These developments illustrate how political and policy contexts—shaping media access, messaging, and regulation [94]—directly influence vaccine promotion, public perception [95], and ultimately, uptake.

#### Access

Limited research has delved into the correlation between healthcare insurance coverage and access to primary care, particularly concerning the HPV vaccine [96, 97]. Studies indicate that expanding insurance coverage could potentially boost HPV vaccination rates among adolescent females by as much as 3% [98]. Insurance coverage for the HPV vaccine is not globally uniform [99, 100].

While recognizing the significance of access to primary care providers in enhancing health outcomes and the cost-effectiveness of healthcare delivery, it becomes crucial to diversify access modalities for vaccine programs. A particular focus must be placed on targeting younger age groups when the vaccine is most effective and provides long-term protection. One efficient method of implementing HPV vaccine uptake has been through school-based programs, where parents and children are able to understand its implications and fulfil the vaccine requirement with less frequent dose programming for those younger than 14 years of age [101, 102].

A few studies have examined the long-term efficacy of single-dose, two-dose, and traditional three-dose HPV vaccine regimens as strategies to optimize access [50, 103]. Single-dose schedules may offer a more feasible approach in resource-limited settings, while two- and three-dose regimens provide more robust and longer-lasting protection [43]. However, decisions around dosing strategies are closely tied to a country’s economic autonomy, vaccine manufacturing capacity, and the ability to implement effective vaccination campaigns to ensure uptake and completion of the series.

To bolster HPV vaccination rates, especially in rural areas and among specific populations, national-level vaccination research initiatives are warranted [104, 105]. These initiatives should involve a comprehensive review and utilization of state immunization registries, examination of pharmacy-related laws pertaining to vaccine availability and administration, promotion of school-entry recommended vaccines, and evidence-based media campaigns [106].

#### Religiosity, ethics, and culture

The intersection between religiosity and vaccines is intricate, influenced by numerous factors like religious beliefs, cultural practices, and attitudes toward modern medicine [107]. While many religious traditions do not inherently oppose vaccination, some individuals and communities may express hesitancy or refusal due to religious interpretations or concerns [108].

Religious teachings often intersect with apprehensions about vaccine ingredients, such as those derived from animal products or fetal tissue cell lines, leading to moral or ethical objections. An example of this is ‘Kosher’ requirements for those following Judaism which exclude porcine, a similar requirement in Islam’s ‘Halal’ regulations. Likewise, Hindus do not take animal derivatives such as bovine products and are likely to follow a vegetarian lifestyle which avoids animal products altogether. Vaccines may employ animal-based substances or utilize replicated fetal cell lines in their development [109], which poses a concern for followers of religions who adhere to certain moral and dietary restrictions. The use of human cell lines may also pose a bioethical issue [110, 111].

Moreover, religious worldviews shape perceptions of health and illness, impacting attitudes toward preventive measures like vaccination [112]. Historically, certain religious groups have exhibited vaccine hesitancy, often due to specific teachings or cultural norms [113]. For instance, Christian Scientists have historically favored spiritual healing practices over medical interventions, including vaccination [114]. Similarly, certain ultra-Orthodox Jewish communities have faced challenges with vaccine uptake due to cultural beliefs and suspicions about government healthcare intervention [115]. While Muslims generally agree to health interventions necessary for life preservation and adhering to halal ingredients, HPV-related pathologies may not be prioritized due to their preventive nature, perceived lack of direct life-threatening impact compared to viruses like COVID-19 [116], and inadequate healthcare accommodations [117].

Religious beliefs often emphasize family values and norms around sexual behavior, which can strongly shape perceptions and hesitancy toward the HPV vaccine. Recognizing the diversity within religious communities [118], it is important to consider how bioethical perspectives and cultural traditions intersect with vaccine attitudes. Religious bioethics, for example, may provide a framework for assessing whether values such as fidelity, modesty, and responsible intimacy contribute to lower HPV infection rates, while also ensuring that these values do not undermine herd immunity or impede epidemiological investigations. Several of the articles we reviewed alluded to rural communities as being more culturally traditional [106], with a perception that adherence to family-centered values and restrained intimacy is sufficient protection against HPV and other sexually transmitted infections. In this context, ethics, religiosity, and cultural norms converge as powerful influences on social expectations of what constitutes healthy behavior and preventive practice—sometimes positioned as alternatives to biomedical interventions like vaccination. Urbanization, secularism, and liberalism, by contrast, are perceived differently across contexts, generating tension between upstream global health priorities and downstream community realities [119]. This divergence raises important ethical questions about resource allocation and respect for community autonomy: continuing to promote HPV vaccination without acknowledging these moral frameworks risks both inefficiency and mistrust. A more effective approach may lie in identifying common ground, transparently respecting moral and cultural choices, and framing HPV vaccination not as a challenge to traditional values but as an additional option for those who seek to exercise caution or make different choices in protecting themselves and their families.

## Sociodemographic characteristics of patients

### Indigenous

Indigeneity, as a concept, proves challenging to pin down due to its nuanced nature; it does not always align with the perceptions held by Indigenous peoples themselves. However, despite this ambiguity, Indigenous communities share common experiences and increasingly assert their aspirations for self-determination, particularly concerning healthcare decisions. Many definitions of Indigenous people highlight their status as disadvantaged descendants of pre-colonial or pre-state formation inhabitants of a territory [120]. Despite their historical marginalization, Indigenous populations are often underrepresented in both academic literature and vaccine development research [121]. Nevertheless, concerning the HPV vaccine, Indigenous communities face shared challenges and hesitancies. These include distrust in the healthcare system, fears regarding potential side effects, concerns about introducing the vaccine at an early age and its implications for promiscuity, a preference for traditional medicine, a lack of culturally tailored HPV education programs, and limited access to healthcare services [122].

However, amidst these challenges, there are also identified factors that can positively influence HPV vaccine acceptance and adoption within Indigenous communities. These include healthcare providers offering informed recommendations to Indigenous patients, active community engagement initiatives, and ensuring equitable access to healthcare services [123]. These factors are crucial in addressing the complex issues surrounding HPV vaccine hesitancy within Indigenous populations and working towards improving their health outcomes.

### Gender

Gender emerged in multiple dimensions within our review, including the sex of healthcare providers, the sex of patients, and LGBTQ populations as distinct groups for examining barriers and facilitators of HPV vaccine uptake [124]. Most studies approached the research through gender-specific frameworks, often surveying participants to understand motivations for vaccinating daughters [125]. Some extended beyond this traditional focus, exploring mothers’ perspectives on vaccinating sons [126], fathers’ roles in decision-making [127], and provider recommendations for male patients [128]. Collectively, these findings highlight gender as a key sociodemographic determinant that shapes both individual decisions and provider practices surrounding HPV vaccination.

The findings also underscore the limitations of gender-specific vaccine programs. By targeting primarily females, such programs restrict protection against HPV-related cancers in males, place disproportionate responsibility on females to prevent transmission, and potentially undermine herd immunity, particularly in the context of evolving sexual norms and orientations. Evidence from several countries further demonstrates that gender-neutral HPV vaccination strategies are not only more equitable but also more cost-effective [129], strengthening the case for inclusive vaccination campaigns [130].

### Age and sexual norms

Age is a critical sociodemographic factor in the HPV vaccine landscape. The vaccine is approved for use beginning at age 9, with eligibility extending up to 45 years for females and 26 years for males [131]. For individuals under 18, vaccination decisions typically require parental or caregiver consent, introducing additional layers of influence on uptake. Initiation at age 9 was designed to align with a less crowded national immunization schedule [132]; however, this early age is often perceived as too young for discussions about HPV [133], leading to reduced clinician recommendation [134] and increased parental hesitancy [135]. In contrast, adults older than 26 often receive less consistent provider recommendations and face variable insurance coverage, which further shapes access and utilization [136].

Recent reports indicate a second peak in HPV infection incidence among older women, although this is not fully reflected in prevalence rates [137]. It is noteworthy that healthcare insurance coverage for HPV vaccines is inconsistent among adult age group, despite its effectiveness up to 45 years of age [138–141].

As societal norms evolve and life expectancy increases, individuals, even those in monogamous relationships, may re-enter the dating pool later in life, potentially necessitating protection from HPV infection beyond the age of 45 years. Currently, HPV vaccines are not offered to eligible males [128], let alone those older than 45 years. Despite limited evidence on the efficacy of HPV vaccines in older individuals, there is no known harm in administering the vaccine to this population, particularly if they did not receive it in their younger years and are now at risk due to changing sexual behaviors.

It is important to note that eligible males and females may not be sexually active at the time when the HPV vaccine is typically administered. Discussions about HPV vaccination regardless of sexual activity may serve as an indirect factor in promoting timely vaccination rates and highlighting its role in cancer prevention rather than sexually transmitted disease treatment [142].

Given these considerations, patients may express interest in receiving HPV vaccine coverage beyond the age of 45 years of age. Vaccine trials affirm that prophylactic HPV vaccination is safe and effective in preventing the acquisition of target HPV genotypes at any age, offering potential benefits to all women [143]. However, extending vaccination beyond young women requires careful consideration of cost-effectiveness and available resources, aiming to maximize health benefits for the broader population [144].

## Provider’s characteristics

The review identified multiple potential avenues for HPV vaccine delivery and recommendation, including school nurses [145], pharmacists [146], teachers [147], dentists [148, 149] primary care providers [132], and specialists [150] (e.g., ENT). At the same time, the literature noted provider challenges such as system-related burnout, competing community roles, and persistent knowledge gaps [151].

While many research findings pointed to the need for more information, provider input from one study suggested that parental concerns were more immediate ranging from vaccine safety and appropriate age to lack of awareness about benefits for boys. The COVID-19 pandemic further complicated this landscape by disrupting the previously passive acceptance of vaccines [145]. The recommendation of the HPV vaccine by healthcare providers consistently influences patients’ willingness to accept the vaccine and positively impacts vaccination rates [152]. Some studies indicated that certain characteristics of healthcare providers are associated with a higher likelihood of recommending the HPV vaccine. These include being female [153–156] possessing greater medical knowledge about HPV, and demonstrating heightened cultural awareness.

Providers who share the racial and ethnic background of their patients may better understand their cultural beliefs and preferences, facilitating more effective communication and recommendation of the vaccine [157, 158].

Additionally, older providers and those with extensive medical knowledge about HPV [159] are more likely to recommend the vaccine, possibly reflecting their experience and understanding of the vaccine’s benefits [160]. Cultural awareness among providers is also a significant factor in recommending the HPV vaccine [161], as it enables them to navigate sensitive cultural considerations and tailor recommendations to the specific needs and beliefs of their patients [162].

While providers get their immunization guidelines from the Centers for Disease Control and Prevention (CDC) there appears to be a gap in awareness and the utilization among clinicians regarding these CDC resources, suggesting that outreach efforts may not be reaching their full potential. Studies indicated a prevalence of provider hesitancy pertaining to inadequate knowledge, low vaccine confidence, and suboptimal uptake themselves [163]. Providers express a need for additional information specifically concerning the safety and efficacy of the HPV vaccine, indicating a desire for more elaborate and current resources in this area [164, 165].

## Discussion

This review provides a thematic analysis of the current dynamics of HPV-related cancers, revisiting the gaps in vaccine uptake, public health considerations, implementation strategies, and sociocultural factors influencing vaccine acceptance and healthcare delivery in the post-COVID-19 era. HPV-related cancers disproportionately affect populations worldwide, and while HPV vaccination programs have the potential to eliminate these cancers, global challenges and societal hesitancy have hindered progress toward this goal for over a decade.

Geopolitical dynamics play a pivotal role in shaping vaccine administration and uptake, with stark disparities evident between high-income and low- to middle-income countries. The COVID-19 pandemic further accentuated these disparities, underscoring the need for equitable vaccine distribution and global solidarity in addressing public health crises. Despite the intricate interplay between geopolitics and vaccine delivery, clinicians are expected to push the safe and effective administration of vaccines without delving deeply into the geopolitical forces influencing their availability [166, 167]. However, as patients become more informed and engage in discussions about vaccine-related issues, healthcare professionals must be prepared to navigate complex geopolitical discussions and address concerns regarding equitable vaccine access and distribution [168].

Middle-income countries’ decisions to adopt such vaccines may reflect aspirations for modernization and adherence to evidence-based healthcare paradigms predominantly shaped by Western biomedical perspectives. Inherently, the HPV vaccine’s actual focus is preventing a sexually transmitted infection rather than merely targeting cervical cancer prevention which may complicate its adoption, and spark debates surrounding its campaign’s transparency, sexual health education, gender politics, and ethical considerations [34]. Religious and cultural considerations intersect with vaccine attitudes, highlighting the need for culturally competent interventions and engagement strategies. The absence of religiosity in healthcare policy and discourse can hinder engagement with religious bioethics platforms and evidence-based interventions aimed at religious communities. State’s religiosity was found to be associated with low vaccination rate regardless of the religious affiliations of its constituents [169].

Sociodemographic characteristics, including Indigenous status, gender, and age, influence HPV vaccine perceptions and access, necessitating tailored approaches to address disparities. There is limited literature on the necessity of HPV vaccines for individuals practicing low-risk sexual behaviors and on the prevalence of HPV infection among them [170, 171].

Practitioners’ characteristics, notably their recommendations, significantly influence patients’ vaccine acceptance. Overall, higher trust in doctors and healthcare providers correlates with increased HPV vaccine uptake among Black populations and Spanish-speaking parents of adolescent children in the U.S. Conversely, a lack of trust is linked to lower intentions to vaccinate and a preference for advice from healthcare providers of the same ethnicity. This highlights the significance of representation within healthcare systems as a strategy to reduce mistrust.

However, gaps in awareness and utilization of CDC resources among clinicians pose challenges, emphasizing the need for targeted outreach and deliberate dissemination.

## Limitations

This review has several limitations. Although we expanded our search to include both PubMed and Embase, we limited our selection to peer-reviewed articles written in English or translated into English, which may have excluded relevant studies published in other languages or regions. These limitations may have affected the review’s findings by underrepresenting research from non-English-speaking regions or contexts with unique cultural, ethical, or policy perspectives. We also found a major gap in the literature examining religiosity and ethics (i.e.; less than 10 papers explored the effects of religion/religiosity as a factor influencing HPV vaccine uptake). Additionally, given the ongoing nature of the COVID-19 pandemic, the review may not fully capture the most recent developments in vaccine distribution, hesitancy, or health system responses. Future research could strengthen the evidence base by including a broader range of databases, languages, and search terms, and by integrating emerging data as the pandemic continues to evolve.

## Conclusion

The HPV vaccine represents a cost-effective cornerstone in the prevention of HPV-related cancers. Maximizing its effectiveness demands a comprehensive strategy encompassing clinical interventions, public health initiatives, inclusive research, and socio-cultural awareness. By ensuring fair vaccine accessibility, tackling healthcare biases, acknowledging historical trauma and medical distrust, and bolstering healthcare provider knowledge, considerable progress can be achieved in alleviating the impact of HPV-related illnesses and advancing health equity worldwide. This assessment offers crucial perspectives to inform forthcoming research, policy formation, and clinical approaches in the ongoing fight against HPV infections and their repercussions.

## Future directions

Moving forward, expanding cost-effective vaccine programs targeting areas of high disease burden, particularly gender-neutral campaigns targeting adolescents and adults, as well as examining its intersection with religious bioethics, represent crucial avenues for advancement. Additionally, acknowledging the global post-pandemic trauma and encourage independent procurement of HPV vaccines at a lower cost for low- and middle-income countries. This approach prioritizes the unique needs of diverse populations rather than imposing uniform recommendations, fostering inclusivity and effectiveness in HPV prevention strategies.

## Data Availability

The research papers cited in this review can be accessed publicly online through the PubMed and the Embase database. A select number of articles are only accessible through academic institutions which the authors are affiliated with (University of Waterloo and the University of California San Francisco).

## Appendix

**Table A1:** Search terms used for PubMed and embase. The term “[MeSH]” was replaced with “/exp” for embase

“Papillomavirus Vaccines”[MeSH] OR “HPV vaccine” OR “human papillomavirus vaccine”
“Religion and Medicine”[MeSH] OR religion OR religious OR faith
OR “Muslims”[MeSH] OR Muslim OR Islamic
OR “Christianity”[MeSH] OR Christianity OR Christians
OR “Jews”[MeSH] OR Jew OR Jewish OR Judaism
OR “Indigenous Peoples”[MeSH] OR Indigenous
OR politics[MeSH] OR political OR geopolitical
OR “Prejudice”[MeSH] OR “Healthcare Disparities”[MeSH] OR “healthcare discrimination”
OR “Trust”[MeSH] OR “medical mistrust”
OR “COVID-19 Vaccines”[MeSH] OR “COVID vaccine” OR pandemic
OR “Ethics”[MeSH] OR ethics
OR “Healthcare Disparities”[MeSH] OR “health disparities”
OR “Economics”[MeSH] OR “country income”
OR “Health Personnel”[MeSH] OR “healthcare provider”
OR “Aged”[MeSH] OR “older age”
OR “Sex Factors”[MeSH] OR “gender difference”
OR “Social Determinants of Health”[MeSH] OR “Social Determinants of Health”
OR sociocultural OR “sociocultural factors”
OR “Health Equity”[MeSH] OR “Health Equity”
OR culture[MeSH] OR cultural
OR “Health Knowledge, Attitudes, Practice”[MeSH] OR “vaccine uptake”
